# Modern Medico-Psychology and Psychiatry

**Published:** 1894-03-31

**Authors:** T. S. Clouston

**Affiliations:** Lecturer on Mental Diseases, Edinburgh University, and Physician Superintendent Royal Asylum, Morningside


### Modern Medico-Psychology and Psychiatry.

By T. S. Clouston, M.D., Lecturer on Mental Diseases, Edinburgh University, and Physician Superintendent Royal Asylum, Morningside.

### II.—LEGISLATIVE AND STATE MEASURES AND THEIR EFFECTS.

The modern period of legislation for the benefit of the mentally diseased may be said to begin in 1814, when a Committee of the House of Commons brought into the light of day the neglect, the abuses, and the shocking cruelties of the existing asylums for the insane, which were mere "mad houses" or places of detention. Acts for the remedy of those abuses, and for the provision of real hospitals for treatment did not follow at once. One such—largely ineffectual—was passed in 1819; another followed in 1828, and another in 1842. This last provided for the appointment of a really efficient Commission of investigation into the facts, whose report of 1844 is a land mark in the history of mental disease in Great Britain. As Dr. Hack Tuke, in his "History of the Insane in the British Islands," says: "It constitutes the Doomsday Book of all that concerns institutions for the insane at that time." There were 16,821 rate-supported insane in England and Wales then, now there are 90,000. The population has doubled in that time, no doubt, but the increase of the insane known and under proper care has been more than fivefold. What is the real meaning of this? The increase in numbers of those known to suffer from mental disease in these fifty years must in no wise be taken to represent a correspondingly increased liability to the complaint. The most careful observers hesitate about coming to the conclusion that there has been any real increase at all in fact in the number of those who have become insane during the past fifty years more than is accounted for by the increase of population. The real meaning of these figures, therefore, is this, that in 1844 there were only 16,821 of the 45,000 insane then existing in England under any sort of treatment and care, while the legislation that followed that report and sprang out of it has brought care and treatment of a far better kind than any that existed for the richest in 1844 within the reach of the poorest. In this way it is the best proof of the necessity for the Acts of 1845 and of their success. It is analagous to what would have taken place if, in 1850, when there were few hospitals for infectious diseases, there had been on record 10,000 fever cases, while in 1900, when each city had one, there should be 100,000 such cases. What would be thought of the inference that fevers had increased tenfold in the fifty years? In 1845 Lord Shaftesbury, then Lord Ashley, succeeded in passing those most philanthropic statutes the two Lunacy Acts for the maintenance at the public expense, and for the proper care and treatment of all those suffering from mental disease in every county in England. One of those Acts established a permanent Commission to visit and report on every hospital for the insane. That Commission consisted then of six paid members, three doctors and three barristers, and its numbers have not been increased since, though it has five times the work to do. The medical profession generally was of opinion in 1845, and has been so ever since, that all the ordinary visiting and inspection of patients should have been relegated to medical commissioners, while a first-rate lawyer should, along with the Secretary, have sat at the Board and given advice on all legal matters. Mr. Wakley, in the *Lancet*, protested vigorously at the time against the arrangement; and as soon as the medical profession is strong enough in Parliament to assert its just and reasonable claims, no doubt the present arrangements will be altered, and law and medicine will be assigned their proper duties on the Commission, so that the insane may receive the full benefits each is capable of conferring on them.

The effect on the welfare of the insane in England of the labours of the Lunacy Commissioners has been quite incalculable. The public have no idea of it at all. Their ideals have been high; their action has been ceaseless, energetic, and fearless, yet as little irritating as possible to the local authorities. One of their functions has been a steady education of the public mind on the subject of mental disease. Whenever it found a specially energetic and original minded physician to an asylum it backed him up; it held him up as an example to others; it learned his new methods and ideas and spread them elsewhere. It preached the gospel of good food, fresh air, absolute cleanliness, cheerful conditioning, and suitable employments and amusements to all the mentally afflicted. It was, perhaps, not quite scientific or psychological enough; it struck one that the barrister Commissioners especially would have been none the worse of a more intimate acquaintance with medico-psychology and psychiatry. Yet the robust English common sense, the fairness and earnestness of the Commission, made its work effectual and universally respected. The English asylum became the model for the world within ten years after the establishment of the Commission over which Lord Shaftesbury presided as unpaid Chairman for over forty years till his death.

Every man and woman of ordinary sense, not to speak of every good doctor, knows now the good effect on most diseases, especially nervous diseases, of right conditioning of the whole life of the invalid. Such right conditioning antagonises most of those diseases far more effectually than medicine or direct therapeutics in most cases, and in all cases helps the direct action of medicines. It is truly and directly a scientific means of cure. The latest scientific investigations show that a bright colour looked at will increase the circulation of blood in the brain; that a cheerful emotion produced by good music or by a dance will stimulate innervation and digestion, and promote the nutrition of the whole body; that a mental cause will produce a bodily effect, and a bodily cause will produce a mental effect; and that the stomach is the pivot round which the whole man often turns. This sound and common-sense system of therapeutics the English Commission found out in regard to insanity and sedulously preached. They realised, too, most keenly, as we all come to realise when we gain experience enough, that the subjects of mental disease, to be cured, and prevented from getting worse when incurable, need an extraordinary and constant output of mind, volition, and feeling on the part of their guardians. Left to themselves they tend to go down to lower levels of a-socialism and of degradation of habits, and general worsening of psychological condition. Their mental dissolution tends to deepen, if not arrested by active measures of sympathy and mental exertion.

(*To be continued.*)

